# 

**Published:** 1887-06-18

**Authors:** 


### The Hospital.

**An Institution, Family, and Parochial Journal of Hospitals, Asylums, and all Agencies for the Care of the Sick, Criticism and News.**

SATURDAY, JUNE 18TH, 1887.

### Notice to Advertisers.

*To insure prompt insertion, all Advertisements for* THE HOSPITAL *must be sent in direct to the Advertising Agent, Mr. A. P. Watt,* 2, *Paternoster Square, E.C.*

### The Literary Prize.

A PRIZE of **Two Guineas** will be given every month for the best story or incident based upon hospital, or asylum, or student life, or any incident connected with the professions of medicine or nursing.

### THE CHILDREN'S CORNER.

**Third Quarterly Prize Competition.**
Began 2nd Apr., 1887; ends 25th June, 1887.

**Puzzles—Twelfth Week.**

No. 47. NATURAL HISTORY ACROSTIC. 1. The caroller of the skies. 2. The sacred bird of the Egyptians. 3. The songster of the night. 4. A bird which feeds on the grubs in nuts. 5. The king of birds. 6. An early warbler. My initials give the name of a well-known bird.

```
      *
    . * .
  . . * . .
* * * * * * *
  . . * . .
    . * .
      *
```

No. 48. DOUBLE MESOSTICH. 1. A bird. 2. A play upon words. 3. An instructive fiction. 4. My whole. 5. A flowering shrub. 6. A river in England. 7. A vowel.

No. 49. FRENCH PUZZLE. Explain the following sentence

Mazarin fert frances
La France a mille.

No. 50. GEOGRAPHICAL TRANSPOSITION.

I can ne'er match so rich a gown,
But in a manufacturing town.

**Letters—Twelfth Week.**

The Letter-subject for next week will be "Haymaking."

**Results of Tenth Week—Puzzles.**

No. 39. GEOGRAPHICAL CHARADE. Ger-many= Germany.

No. 40. ARITHMETICAL PUZZLE. Only Alsie and Skye have succeeded in solving this puzzle correctly as follows:—XI, II = XIII.

No. 41. SQUARE WORD.

R O S E
O F F A
S O U R
E A R S

No. 42. VEGETABLE ANAGRAMS. Most of my young friends have discovered the "Onions" and "Potatoes."

*All right*—Alsie, Skye; *Three right*—Nordisa, A. G. S. McCulloch, Scrooge, Dot, R. Epton, A. C. K.; *Two right*—Bohemian Girl, Mikado, Joe, Gom, Umslopogaas, Tim.

**Letters.**

The subject for last week's letter does not seem to be a popular one with my little correspondents, and I am surprised to have only received letters from girls. Where are the boys? At this time of the year some of them must surely be commencing to make a collection of Birds' Eggs, and I hoped that they would have much that was interesting to tell me on the subject. Mikado writes that the Emu eggs in the Colonial Exhibition were amongst the most beautiful she has ever seen. I quite agree with her; they were one of the most interesting and attractive objects in the Western and Australian courts. Skye seems to find the subject of Birds' Eggs a very uncongenial one, and it may ease her tender heart to know that sometimes eggs are obtained without robbing nests, for Bohemian Girl tells me that she once found a very pretty Blackbird's egg *under* a hedge! I hope that my next week's subject, "Haymaking," may meet with general favour. Summer has come at last, and you all love a day in the hayfield. If you have never had this delight, tell me how you would like to spend a day in the country.

One mark each:—Mikado, Bohemian Girl, Skye.

**Notices to Correspondents.**

BOHEMIAN GIRL.—Many thanks for the riddle; it is rather a warm one for this time of the year. I will make use of it when the days grow cold again.

Answers must be addressed to the "PUZZLE EDITOR," THE HOSPITAL, Norfolk House, Norfolk Street, Strand, W.C., not later than Thursday next. The "Children's Corner" Coupon (see advertisement pages) must be enclosed.

### AMUSEMENTS WITH PROFIT.

### PRIZE COMPETITIONS.

**Quarterly Prize Competition.**
Began 23rd April; ends 16th July.

**No. 17. Double Acrostic.**

1. A Roman emperor, tho' of humble birth,
   Possessed of dignity and sterling worth;
2. A country in the East, where you may find
   Both worldly goods and riches for the mind;
3. Next take a piece of wood which is of use to some,
4. And then a plant a man enjoys when he is with his chum;
5. An extract follows, not taken from a book,
   'Tis rather to be found with druggist and with cook;
6. Imagine then a contest, and then a Christian name
7. Belonging to a man of architectural fame;
   To close this varied list, from Greece's classic land
8. A lyric poet comes, and waves his magic wand.

The *initials* and *finals*, if now you will take,
Two persons of note they will certainly make.

**No. 18. Charade.**

Through my first for ever flow
Sounds of joy and sounds of woe;
In my second, freshly made,
Thousands year by year are laid.
In my whole we never jest;
Prayers are offered, vows expressed.

**Answers.**

No. 13. DOUBLE ACROSTIC.

| | | |
|---|---|---|
| A | l | B |
| B | eha | R |
| E | ssequib | O |
| R | enfre | W |
| N | ewto | N |
| E | the | R |
| T | ahit | I |
| H | eidelber | G |
| * | * * * | * |
| Y | ang-tze-Kian | G |

The Puzzle-Editor much regrets that the last light, "One of the largest rivers in the world," was inadvertently omitted from the above acrostic, and therefore credits all competitors with it.

No. 14. DEFINITION OF HEROISM.

All our competitors, with one exception, have chosen prose in which to define the noble deeds effected by Heroism. Most of the answers are clever and to the point, particularly the one in verse.

Best answers have been received from Aralc, Mater, K. M., Rev. J. E. L. N., Letheador, Gretta, Ellice.

**Notices to Correspondents.**

BROADWATER.—Sorry your definition arrived too late to be credited.

H. B.—Has omitted to observe the rule, "that full name and address must in all cases be given."

CANICE.—Only one side of the paper should be written on.

Answers must be addressed to THE PRIZE EDITOR, AMUSEMENTS WITH PROFIT, THE HOSPITAL, Norfolk House, Norfolk Street, Strand, W.C., not later than the *first* post on Friday next. The full name and address must in all cases be given, but a *nom de plume* may be added for publication. Only one side of the paper should be written on. The "Amusements with Profit" Coupon (see advertisement pages) must be enclosed with replies.

### THE IN- AND OUT-PATIENTS' HOSPITAL DIARY.

This Diary is so arranged as to make some portion of it appear every week, and the whole in each monthly part. It has been very difficult to compile, and corrections will at all times be gratefully received. It has been suggested that similar information about the larger Provincial and Scotch Hospitals would be useful to many readers. We should be glad to hear if this is felt to be the case.

### GENERAL HOSPITALS.—*continued.*

| Hospitals and Terms of Admission. | Physicians, Surgeons, and Medical Officers, with Days and Hours of Attendance. |
|---|---|
| St. Mary's Hospital, Cambridge Place, Paddington, W. Sec., Mr. Pietro Michelli<br>In-patients are admitted by letter of recommendation. Urgent cases and accidents free at all times. Out-patients require a letter. Electrical treatment Tu. and F, 2 | *In-Physicians*, Sir Edward Sieveking, Tu. F. 1.45; Drs. Broadbent, M. Th. 1.45; Cheadle, W. 1.45, S. 9.30<br>*In-Surgeons*, Messrs. Norton, M. Th. 1.45; Owen, Tu. F. 1.45; H. Page, W. S. 1.45<br>*Out-Physicians*, Drs. D. B. Lees, W. S.; S. Phillips, M. Th; R. Macguire, Tu. F. Hour, 1.30<br>*Out-Surgeons*, Messrs. W. Pye, M. Th.; A. J. Pepper, Tu. F.; A. Q. Silcock, W. S. Hour, 1.30 |
| St. Thomas's Hospital, Westminster Bridge Road, S.E. Steward, Mr. F. Walker<br>Accidents and emergencies free at all times. Other cases by Governor's letter, but, where unobtainable, application should be made to the Steward by letter, or personally between 10 and 4. Vaccination on W. at 11.30 | *In-Physicians*, Drs. Bristowe, Tu. F.; Stone, M. Th.; Ord, M. Th.; J. Harley, Tu. F.; Gervis, Tu. F. Hour, 2<br>*In-Surgeons*, Messrs. Sydney Jones, Tu. F.; Croft, M. Th.; Sir W. MacCormac, M. Th. Hour, 2<br>*Out-Physicians*, Drs. Payne, Tu. F.; Sharkey, M. Th.; Gulliver, W. S. Hour, 1.30<br>*Out-Surgeons*, Messrs. Clutton, Tu. F.; Anderson, W. S.; Pitts, M. Tu. Hour, 1.30 |
| Tottenham Training Hospital, Tottenham, N. Director, Dr. Laseron<br>Free. Accidents and emergencies admitted at all times | *In-Physician*. Dr. Rasch, Tu. F. 3 to 5<br>*In-Surgeons*, Drs. Lichtenberg, M. Th. 3 to 5; E. H. May, M. Th. 3 to 5<br>*Out-Surgeon*, Dr. Lloyd Smith, M. W. 11 to 2; *Men* only, W. 7 to 8 p.m. |
| University College Hospital (or North London), Gower Street, W.C. Sec., Mr. N. H. Nixon<br>Accidents and emergencies free. All other cases by Governor's letter of recommendation | *In-Physicians*, Drs. W. Fox, Tu. Th. 2, S. 9.30; Ringer, M. W. F. 1.30; Bastian, M. 2.30, W. 10, F. 2.30; F. Roberts, Tu. 1.15, Th. 1.30, S. 9.30<br>*In-Surgeons*, Messrs. B. Hill, Tu. 2.15, W. 2, F. 2.15; C. Heath, M. W. F. 2; M. Beck, Tu. Th. S. 2<br>*Out-Physicians*, Drs. Gowers, W. S.; Poore, Tu. F.; Barlow, M. Th. Hour, 1.30<br>*Out-Surgeons*, Messrs. Barker, M. Th.; Godlee, Tu. F.; Horsley, W. S. Hour, 1.30 |
| West London Hospital, Hammersmith Road, W. Sec. and Supt., Mr. R. J. Gilbert<br>Accidents and emergencies free at all times. Other cases require a letter of recommendation. New cases must attend at 1.30 | *In-Physicians*, Drs. G. Rogers, D. W. Hood, F. Drewitt<br>*In-Surgeons*, Messrs. C. Keetley, F. Edwards, W. B. Clarke<br>*Out-Physicians*, Drs. Herringham, M. Th.; Ball, Tu. F.; S. Taylor, W. S. Hour, 2<br>*Out-Surgeons*, Messrs. Ballance, Tu. F.; Weiss, M. Th.; Wainewright, W. S. Hour, 2 |
| Westminster Hospital, Broad Sanctuary, S.W. Sec., Mr. S. M. Quennell<br>Accidents and urgent cases free at all times. Other cases require a letter of recommendation | *In-Physicians*, Drs. Sturges, M. Th. S.; Allchin, M. Tu. F.; Donkin, Tu. W. S. Hour, 1.30<br>*In-Surgeons*, Messrs. Cowell, M. Tu. Th.; Davy, M. Tu. F.; Macnamara, M. W. S. Hour, 1.30<br>*Out-Physicians*, Drs. De H. Hall, M. Th.; A. H. Bennett, Tu. F.; Murrell, W. S. Hour, 1.30<br>*Out-Surgeons*, Messrs. Cooke, M. Th.; Bond, Tu. F.; Barrow, W. S. Hour, 1.30 |

### II.—SPECIAL HOSPITALS.

### CANCER.

| | |
|---|---|
| Cancer Hospital, Brompton, S.W. Sec., Mr. W. H. Hughes<br>Entirely free to both in- and out-patients | *In- and Out-Surgeons*, Drs. A. Marsden, W.; H. Snow, M.; F. A. Purcell, F.; Messrs. F. B. Jessett, W.; C. Stonham, Th.; C. Jennings, Tu.; W. H. Elam, S. Hour, 2 |
| London Hospital, Whitechapel Road, E. | Daily, 1.30. *See* General Hospitals |
| Middlesex Hospital, Berners Street, W. Admission free | *In-Surgeons*, Messrs. Hulke, Lawson, and Morris<br>*Out-Surgeon*, Mr. Morris, Th. 1.30 |
| St. Saviour's Hospital, Osnaburg Street, N.W. Sec., Mr. E. Poitiers<br>By Governors' letters | *In- and Out-Physician*, Dr. A. Kennedy, 10.30 and 4 daily |

### CHILDREN'S DISEASES.

| Hospitals and Terms of Admission. | Physicians, Surgeons, and Medical Officers, with Days and Hours of Attendance. |
|---|---|
| Alexandra Hospital for Children, 18, Queen Sq., W.C. Sec., Mrs. Marsh<br>For hip disease | *Physician*, Dr. Steavenson<br>*Surgeons*, Messrs. H. Marsh Tu. F.; A. Bowlby, M. Th. Hour, 4.30<br>(No out-patients) |
| All Saints' Children's Hospital, 4, Margaret Street, W.<br>For chronic joint diseases | *Surgeon*, Mr. N. Smith<br>(No out-patients) |
| Belgrave Hosp. for Children, 77 & 79, Gloucester Street, Pimlico. Hon. Secs., Capt. Stopford, and Rev. J. Storrs<br>By letters; accidents free | *In- and Out-Physicians*, Drs. W. Hope and W. Ewart, M. Th. 9<br>*In- and Out-Surgeons*, Messrs. C. T. Dent and Margerison, Tu. F. 9 |
| Charing Cross Hospital, West Strand, W.C. | *Out-Physician*, Dr. Nursby, W. S. 1.30 |
| Cheyne Hospital for Sick and Incurable Children, 46, Cheyne Walk, Chelsea, S.W. Sec., Miss D. Evans | *Physician*, Dr. G. V. Poore<br>*Surgeons*, Messrs. T. P. Bartlett and J. Macready attend alternate days in afternoons. (No out-patients) |
| East London Hospital for Children and Dispensary for Women, Glamis Road, Shadwell, E. Sec., Mr. Ashton Warner<br>By Governor's letter. Urgent cases and accidents are admitted free | *In-Physicians*, Drs. Donkin, M. Th.; Eustace Smith, T. F.; Crocker, F.<br>*In-Surgeons*, Messrs. A. Cæsar, R. W. Parker, W. F.<br>*Out-Physicians*, Drs. Crocker, M.; Dawson Williams, Tu. Th.; Coutts, W. F. Hours, 1 and 3<br>*Out-Surgeon*, Mr. Dunn, Tu. F. 1, 3 |
| Evelina Hospital for Sick Children, Southwark Bridge Road, S.E. Sec., Mr. T. S. Chapman.<br>Free, but Governor's letter takes precedence. In-patients (boys) between 2 and 10, (girls) between 2 and 12 | *In-Physicians*, Drs. F. Taylor and J. Goodhart<br>*In-Surgeons*, Messrs. H. G. Howse and R. C. Lucas<br>*Out-Physicians*, Drs. N. Tirard, M. Th. 9; F. Willcocks, Tu. F. 9<br>*Out-Surgeons*, Messrs. C. J. Symonds, S. 9; G. H. Makins, W. 9 |
| German Hospital, Dalston Lane, Dalston, E. | *In-Physician*, Dr. Rasch, M. Th. 9 |
| Great Northern Central Hospital, Caledonian Rd. N. | *Physicians*, Drs. Murray and Barnes W. 2.30 |
| Grosvenor Hospital for Women and Children, Vincent Sq., S.W. Hon. Secs., Mr. A. J. Ram and Miss Day<br>By letter or payment | *In- and Out-Physicians*, Drs. R. Gibbons and Steavenson, Tu. W. Th. S. 2<br>*In- and Out-Surgeons*, Messrs. Smythe and Parker, Tu. W. Th. S. 2 |
| Hospital for Sick Children, 48 and 49, Great Ormond St., W.C. Sec., Mr. S. Whitford<br>By Governors' letters. If without letter, the case of the applicant is investigated<br>In-patients between 2 and 12; Out-patients under 12 | *In-Physicians*, Drs. W. Cheadle, Tu. F.; Sturges, W. S.; Barlow, M. Th. Hours, 9 to 11<br>*In-Surgeons*, Messrs. H. Marsh, W. S.; E. Owen, W. S. Hours, 9 to 11<br>*Out-Physicians*, Drs. R. J. Lee and Abercrombie, M. Th.; Lubbock and Money, Tu. F. Hours, 8.30 to 10<br>*Out-Surgeons*, Messrs. Morgan and Pitts, W. S. 8.30 to 10 |
| King's College Hospital, Portugal Street, W.C. | *In- and Out-Physician*, Dr. T. C. Hayes, W. F. 1 |
| Middlesex Hospital, Berners Street, W.<br>Free. Children under 3 | *Out-Physician*, Dr. Duncan, W. S. 1.30 |
| North Eastern Hospital for Children, Hackney Road, E. Sec., Mr. A. Nixon<br>By free ticket or small payment | *In- and Out-Physicians*, Drs. W. Cayley, F. Turner, C. Semple, and W. Pasteur, M. W. Th. S. 1.30<br>*In- and Out-Surgeons*, Messrs. Waren Tay and Pollard, M. 1.30, F. 9 |
| Paddington Green Children's Hosp., Paddington Green. Sec., Mr. W. H. Pearce<br>Admission free; boys under 12; girls under 14 | *In- and Out-Physicians*, Drs. T. Lauder Brunton, Ogilvie, Tu. F.; G. Laycock, M. Th.; S. Phillips, W. S. Hour, 9.15<br>*In- and Out-Surgeons*, Messrs. W. W. Cheyne, M., S. Boyd, F. Hour, 9.15 |
| Royal Hospital for Children and Women, Waterloo Bridge Road, S.E. Sec., Mr. R. G. Kestin<br>Out-Patients pay 1*d.* on each attendance. Boys not admissible, as out-patients, over 12, or, as in-patients, over 8 | *In-Physicians*, Drs. Park, M. Th.; Gulliver, Tu. F.; Price, W. S. Hour, 2<br>*In-Surgeon*, Mr. W. H. A. Jacobson, M. Th. Hour, 2<br>*Out-Physicians*, Drs. Park, M. Th.; Dakin, W. F.; Hadden, Tu. S. Hour, 2<br>*Out-Surgeon*, Mr. E. O. Day, W. 2 |
| St. Mary's Hospital, Cambridge Place, Paddington | *Out-Physician*, Dr. M. Handfield Jones, M. Th. 1.30 |
| St. Thomas's Hospital, Westminster Bridge Road, S.E | *Out-Physician*, Dr. Cory, W. S. 1.30 |

### The In- and Out-Patients' Hospital Diary.

### CHILDREN'S DISEASES.—*continued.*

| Hospitals and Terms of Admission. | Physicians, Surgeons, and Medical Officers, with Days and Hours of Attendance. |
|---|---|
| Samaritan Free Hospital for Women and Children, Lower Seymour Street, W.; Branch, 1, Dorset St., W. Sec., Mr. G. Scudamore. In-patients must be between 2 and 12. Out-patients Department (free) is at Edward's Mews, Duke Street, W. | *In-Physician*, Dr. W. H. Day. Daily, 2 *In-Surgeon*, Mr. W. A. Meredith. Daily, 2 *Out-Physicians*, Drs. M. Prickett and Amand Routh, W. S. 1.30 *Out-Surgeons*, Messrs. W. A. Meredith and J. D. Malcolm, M. Th.; A. H. G. Doran and W. S. A. Griffith, Tu. F., 1.30 |
| Victoria Hosp. for Children, Queen's Road, Chelsea, S.W. Sec., Capt. W. C. Blount, R.N. Age of boys, 2 to 12 years; age of girls, 2 to 16. Admission for in-patients by subscriber's letter. Accidents and urgent cases free at all times | *In-Physicians*, Drs. J. Evans, M. Th. 2; Ridge-Jones, Tu. F. 2 *In-Surgeons*, Messrs. Pick, Tu. F. 9.15; Clutton, M. Th. 4.30 *Out-Physicians*, Drs. A. Venn, W. S. 9.30; C. Fox, M. Th. 1.30; F. D. Drewitt, Tu. F. 9; J. Philpot, M. Th. 9 *Out-Surgeons*, Messrs. F. Churchill, Tu. F. 10; W. Pye, M. Th. 9.30 |
| West London Hospital, Hammersmith rd, W. | Daily, 2. *See* General Hospitals |

### CONSUMPTION AND DISEASES OF THE CHEST.

| | |
|---|---|
| City of London Hospital for Diseases of the Chest, Victoria Park, E. Sec., Mr. T. Storrar Smith By subscriber's letter available for 6 weeks. | *In-Physicians*, Drs. J. Thorowgood, S.; E. Smith, M.; J. Fothergill, W.; S. West, F.; G. A. Heron, W.; V. D. Harris, Tu. Hour, 2 *Out-Physicians*, Drs. V. D. Harris, Tu.; J. A. Ormerod, Th.; E. C. Beale, Tu. F.; B. Fenwick, W. S.; A. Money, W. S.; L. E. Shaw, M. Th. Hour, 2 |
| Hospital for Consumption, Fulham road, Brompton, S.W. Sec., Mr. H. Dobbin Admission by letter of recommendation, but, where unobtainable, application may be made to the Secretary at the Hospital. | *In-Physicians*, Drs. E. S. Thompson, M. Th.; C. T. Williams, M. Th.; R. D. Powell, Tu. F.; J. Tatham, W. S.; R. E. Thompson, W. S.; F. T. Roberts, Tu. F. Hours, 2 to 4 *In-Surgeon*, Mr. R. J. Godlee, F. *Out-Physicians*, Drs. T. H. Green, J. M. Bruce, M. Th.; J. K. Fowler, W. S.; P. Kidd and C. Y. Biss, Tu. F.; T. D. Acland, W. S. Hour, 12 |
| North London Hospital for Consumption and Diseases of the Chest, Mount Vernon, Hampstead, N. Sec., Mr. L. F. Hill Admission by subscriber's letter available for 6 weeks. | *In-Physicians*, Drs. A. Evershed, Tu.; B. O'Connor, F.; D. Pidcock, F. Hour, 10.30 *Out-Physicians*, Drs. B. O'Connor, D. Pidcock, J. E. Squire. Daily, 2 (attend at Out-patients' Branch, 216, Tottenham Court Road, W.) |
| Royal Hospital for Diseases of the Chest, 231, City Rd., E.C. Sec., Mr. John J. Austin By Governor's letter, available for six weeks | *In-Physicians*, Drs. Hensley, W.; Gilbart-Smith, M.; Finlay, Th. *In-Surgeon*, Mr. A. Pearce-Gould, M. Tu. W. Th. F. 2, S. 9 *Out-Physicians*, Drs. White, Tu. F.; Pringle, M. Th.; O. Browne, W. S.; A. Davies, M. F.; H. Habershon, W. S.; E. Stewart, Tu. Th. Hour, 1; S. 9 |
| Royal National Hosp. for Consumption and Diseases of the Chest, Ventnor. Offices, 34, Craven St., Strand, W.C. Sec., Mr. E. Morgan Admits only incipient cases by Governor's letter. | *In-and Out-Physicians*, Drs. J. G. Sinclair Coghill, and R. Robertson attend daily (at Ventnor) on out-patients at 11; on in-patients at noon *In- and Out-Surgeons*, Messrs. Whitehead, Williamson |

### EAR CASES.

| | |
|---|---|
| Central London Throat and Ear Hosp., Gray's Inn Rd., W.C. Sec., Mr. R. Kershaw Free, but when able, a small payment is expected | *In- and Out-Surgeons*, Mr. Lennox Browne, M, Th. 2.30; Dr. Orwin, W. S. 2.30; Dr. Grant, Tu. 5, Th. 2.30; Messrs. P. S. Jakins, M. W. 2.30; F. 5; T. Carmalt Jones, M. Th. S. 2.30 |
| Charing Cross Hospital, West Strand, W.C. | *In- and Out-Surgeon*, Mr. Cantlie, F. 9 |
| Great Northern Central Hospital, Caledonian Rd, N. | *In- and Out-Surgeon*, Mr. A. E. Cumberbatch, W. 9 |
| Guy's Hospital, St. Thomas's Street, S.E. | *In- and Out-Surgeon*, Mr. Purves, Tu. F. 12.30 |
| Hospital for Diseases of the Throat, Golden Square, W. Sec., Mr. W. T. Sharp | *Physicians*, Drs. Wolfenden, Tu. F. 2.30; Macdonald, Tu. F. 6.30; Bond, W. S. 2.30 *Surgeon*, Mr. T. M. Hovell, M. Th. 2.30 |

### EAR CASES—*Continued.*

| Hospitals and Terms of Admission. | Physicians, Surgeons, and Medical Officers, with Days and Hours of Attendance. |
|---|---|
| King's College Hospital, Portugal Street, W.C. | *In- and Out- Surgeon*, Mr. Pritchard Th. 2.30 |
| London Hospital, Whitechapel Road, E. | *In- and Out-Surgeons*, Dr. Woakes, Mr. T. M. Hovell, S. 9 |
| Middlesex Hospital, Berners Street, W. Free. | *Out-Surgeon*, Mr. Hensman, Tu. 9 |
| National Hosp. for the Paralysed, etc., Queen Sq., W.C. | *In- and Out-Surgeon*, Mr. A. E. Cumberbatch, W. 2 |
| North West London Hospital, 18, Kentish Town Rd. | *Out-Surgeon*, Mr. Collier, M. 1.30 |
| St. Bartholomew's Hospital, Smithfield, E.C. | *Surgeon*, Mr. Cumberbatch, Tu. F. 2 |
| St. George's Hospital, Hyde Park Corner, S.W. | *In- and Out-Surgeon*, Sir W. B. Dalby, Tu. 1.30 |
| St. Mary's Hospital, Cambridge Place, Paddington, W. | *In- and Out-Surgeon*, Mr. G. P. Field, M. Thur. 3 |
| St. Thomas's Hospital, Westminster Bridge Road, S.E. | *Out-Surgeon*, Mr. Clutton, M. 1.30 |
| University College Hospital, Gower Street, W.C. | *In- and Out-Surgeon*, Mr. Barker, Th. 9 |
| Westminster Hospital, Broad Sanctuary, S.W. | *In- and Out-Surgeon*, Mr. Barrow, Tu. F. 9 |

### EYE DISEASES.

| | |
|---|---|
| Central London Ophthalmic Hosp., 238a, Gray's Inn Rd., W.C. Sec., Mr. G. H. Leah Admission free | *Surgeons*, Messrs. T. Archer, G. Abbott, W. Charnley, W. Jessop, E. Clarke, J. R. Kemp and Granville Barker (House Surgeon). Daily, 1 |
| Evelina Hosp. for Sick Children, Southwark Bridge Rd. | *Surgeon*, Dr. W. Brailey, Th. 1 |
| Great Northern Central Hospital, Caledonian Rd., N. | *Surgeon*, Mr. Hutchinson, Jnr., Tu. F. 9.30 |
| Guy's Hospital, St. Thomas's Street, S.E. | *Surgeons*, Mr. Higgens, Dr. Brailey, Tu. F. 12.30 |
| Hospital for Sick Children, 49, Gt. Ormond Street, W.C. | *Surgeon*, Mr. R. Marcus Gunn, Tu. 2 |
| King's College Hospital, Portugal Street, W.C. | *Surgeon*, Mr. McHardy, M. W. Th. 1.30 |
| London Hospital, Whitechapel Road, E. | *Surgeons*, Messrs. Waren Tay, S. 9; Eve, Tu. 9 |
| Middlesex Hospital, Berners Street, W. | *Surgeon*, Mr. Lang, Tu. F. 9 |
| National Hosp. for the Paralysed, etc. Queen Sq., W.C. | *Surgeons*, Messrs. R. Brudenell Carter, F. 4; R. Marcus Gunn, Tu. 4 |
| North-West London Hosp. 18, Kentish Town Rd., N.W. | *Surgeon*, Dr. W. Collins, F. 3.30 |
| Paddington Green Children's Hosp., Paddington Grn, | *Surgeon*, Mr. W. H. H. Jessop, Th. 9 |
| Royal Free Hospital, Gray's Inn Road, W.C. | *Surgeon*, Mr. Mackinlay, M. F. 9 |
| Royal London Ophthalmic Hospital, Blomfield Street, Moorfields, E.C. Sec., Mr. R. J. Newstead Admission free to the poor. | *Surgeons*, Messrs. J. W. Hulke, W. S.; G. Lawson, Tu. F.; J. Couper, Tu. F.; W. Tay, M. Th.; J. Tweedy, Tu. F.; E. Nettleship, W. S.; R. M. Gunn, W. S.; Lang, M. Th. Hours, 8 to 10 |
| Royal South London Ophthalmic Hosp., 6, St. George's Circus, S.E. Sec., Mr. C. Comyn Free to the necessitous poor; or by payment | *Surgeons*, Messrs. Watson, McHardy. Daily, 2 |
| Royal Westminster Ophthalmic Hospital, King William Street, W.C. Sec., Mr. T. Beattie-Campbell Poor and accidents free | *In- and Out-Surgeons*, Messrs. Power, M. F.; Frost, M. Th.; Rouse, Tu. S.; Hartridge, Tu. F.; Macnamara, M. Th.; Cowell, W. S.; Juler, W. S. Hour, 1 |
| St. Bartholomew's Hospital, Smithfield, E.C. | *Surgeons*, Messrs. Power, Th. 2; Vernon, Tu. S. 2.30 |
| St. George's Hospital, Hyde Park Corner, S.W. | *Surgeons*, Messrs. B. Carter, Frost, M. Th. 2; F. 1.15; W. S. 2 |
| St. Mary's Hospital, Cambridge Place, Paddington. W. | *Surgeons*, Messrs. A. Critchett, H. Juler, Tu. F. S. 9 |
| St. Thomas's Hospital, Westminster Bridge Road, S.E. | *Surgeons*, Messrs. Nettleship, Tu. Th. F. 1.30; Lawford, M. W. 1.30 |
| University College Hospital, Gower Street, W.C. | *Surgeon*, Mr. Tweedy, M. Tu. Th. F. 2 |
| West London Hospital, Hammersmith Road, W. | *Surgeons*, Messrs. B. Vernon, H. Dunn, Tu. Th. S. 2 |
| Westminster Hospital, Broad Sanctuary, S.W. | *Surgeon*, Mr. Cowell, M. Th. 2 |
| Western Ophthalmic Hosp. 153 and 155, Marylebone Rd., W. Sec., Mr. G. E. Manton. By letter or small payments; free to poor | *Surgeons*, Drs. Miller, M.; Collins, F.; Wheatly, M. Th.; Messrs. Carmal, Jones, W.; Buxton, Tu. F.; Nunn W. S. Hour, 2 |

